# Supplementary figures and images for: IL-4 Augments IL-31/IL-31 Receptor Alpha Interaction Leading to Enhanced Ccl 17 and Ccl 22 Production in Dendritic Cells: Implications for Atopic Dermatitis
Source: Int J Mol Sci. 2019 Aug 20;20(16):4053. doi: 10.3390/ijms20164053 (PMC6719908; doi:10.3390/ijms20164053)

**a**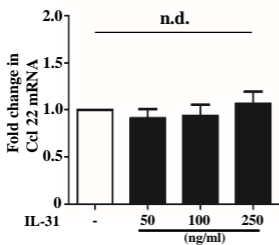**b**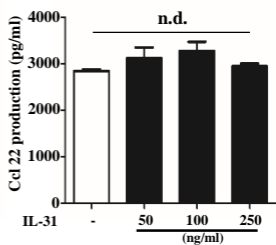

**【Supplemental Figure 1】**

Supplement: Supplementary file 1 [file ijms-20-04053-s001.pdf]
